# Biophysical Constraints on Optimal Patch Lengths for Settlement of a Reef-Building Bivalve

**DOI:** 10.1371/journal.pone.0071506

**Published:** 2013-08-19

**Authors:** Heidi L. Fuchs, Matthew A. Reidenbach

**Affiliations:** 1 Institute of Marine and Coastal Sciences, Rutgers University, New Brunswick, New Jersey, United States of America; 2 Department of Environmental Sciences, University of Virginia, Charlottesville, Virginia, United States of America; University of Sydney, Australia

## Abstract

Reef-building species form discrete patches atop soft sediments, and reef restoration often involves depositing solid material as a substrate for larval settlement and growth. There have been few theoretical efforts to optimize the physical characteristics of a restored reef patch to achieve high recruitment rates. The delivery of competent larvae to a reef patch is influenced by larval behavior and by physical habitat characteristics such as substrate roughness, patch length, current speed, and water depth. We used a spatial model, the “hitting-distance” model, to identify habitat characteristics that will jointly maximize both the settlement probability and the density of recruits on an oyster reef (*Crassostrea virginica*). Modeled larval behaviors were based on laboratory observations and included turbulence-induced diving, turbulence-induced passive sinking, and neutral buoyancy. Profiles of currents and turbulence were based on velocity profiles measured in coastal Virginia over four different substrates: natural oyster reefs, mud, and deposited oyster and whelk shell. Settlement probabilities were higher on larger patches, whereas average settler densities were higher on smaller patches. Larvae settled most successfully and had the smallest optimal patch length when diving over rough substrates in shallow water. Water depth was the greatest source of variability, followed by larval behavior, substrate roughness, and tidal current speed. This result suggests that the best way to maximize settlement on restored reefs is to construct patches of optimal length for the water depth, whereas substrate type is less important than expected. Although physical patch characteristics are easy to measure, uncertainty about larval behavior remains an obstacle for predicting settlement patterns. The mechanistic approach presented here could be combined with a spatially explicit metapopulation model to optimize the arrangement of reef patches in an estuary or region for greater sustainability of restored habitats.

## Introduction

Reef-building marine invertebrates, including oysters, mussels, and corals, form discrete clumps or patches that are elevated above the surrounding sediments. When reefs become degraded, some restoration efforts focus on constructing patches of solid substrates to stimulate larval recruitment and growth [1]. Both the size and roughness of constructed substrate patches may influence the ability of artificial reefs to accumulate new settlers. Larger reefs present a larger settlement target for individual larvae but facilitate settlement over a larger area and may ultimately receive a lower spatially-averaged density of settlers. Rougher reef substrates generate more turbulence, which affects the delivery of larvae to the bed by diffusive mixing and can enhance or reduce settlement depending on larval behavior. Although patch characteristics influence both behavioral and physical processes delivering larvae to the bed, there have been few attempts to quantify the relationship between substrate patch size or roughness and larval settlement. Here we develop a quantitative approach for identifying the characteristics of a habitat patch that will maximize settlement both for individual larvae and for the reef patch.

Eastern oysters (*Crassostrea virginica*) are an ideal model species for investigating how patch characteristics affect settlement success, because oysters form coarse beds or reefs on soft sediments. Natural oyster reefs are 10 s to 1000 s of m long in the prevailing current direction and are found in intertidal to shallow subtidal regions in estuaries and near shore. Natural reefs in North America tend to be smaller and more intertidal in the South Atlantic region and larger and more subtidal in the North Atlantic region [2], [3]. Oyster restoration most commonly involves constructing artificial reefs by depositing patches of artificial or natural substrate on mud or sand flats [1]. These constructed reefs can boost fitness of the metapopulation as a whole if they reduce larval mortality by increasing the probability that individual larvae will land on an oyster reef. For individual reefs to succeed, however, recruitment rates and adult densities must be high enough for shell accretion to outpace shell loss, so that the reef grows rather than becoming degraded or buried [4]–[6]. Maximizing settlement may be particularly important in years of low larval supply. Oyster management could benefit from theoretical guidelines on how the size and shape of constructed reefs impact the success of individual reefs in accumulating recruits at high densities.

Existing guidelines suggest that constructed reefs are more successful if they have high vertical relief and are composed of natural substrates with complex surfaces. Higher artificial reefs have higher spat recruitment and adult growth rates [4], [7], [8], in part because elevation above the seafloor provides some protection from sedimentation and hypoxic bottom waters [9], [10]. Rougher substrates also have higher recruitment rates, perhaps because they provide recruits with interstitial spaces as a refuge from predators [11], [12]. The preferred substrate is oyster shell, which provides more habitat microstructure than smoother, flatter shells and may contain residual chemicals that cue oyster larvae to metamorphose [4], [11], [13]. Although recruitment rates and adult survival vary with reef height and substrate types, few studies have directly addressed how substrate roughness or patch size affect the supply of larvae to a constructed reef.

Here we focus on optimizing the size of a reef using a mechanistic study of the physical and behavioral processes delivering larvae to a reef patch. Competent larvae pass over a reef patch from some starting height and can settle on the first pass only if they reach the bed before they are swept past the reef. The distance traveled before hitting bottom (the “hitting distance”) depends on horizontal advection by currents and on vertical delivery of larvae to the bed by larval behaviors and turbulent mixing [14]. Vertical mixing is enhanced by rough substrates that increase bottom drag and generate turbulence [15], [16]. Turbulent mixing can raise or lower settlement probabilities, depending on larval behavior: ascending larvae have higher settlement probabilities in stronger turbulence, whereas descending larvae have lower settlement probabilities in stronger turbulence [17]. Turbulence and larval behavior are intricately linked in bringing larvae in contact with habitat patches.

The role of turbulence in larval supply is more complex when turbulence itself induces behavioral changes. Some mollusc larvae swim up in calm water but retract the velum and sink in turbulence above some threshold value of the dissipation rate of turbulent kinetic energy 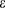 [18], [19]. Eyed oyster larvae have near-zero velocities in calm water, but in strong turbulence (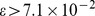 cm^2^ s^-3^) they actively propel themselves downward [20]. Active diving requires energy, and its ecological advantage is unclear because oyster larvae are negatively buoyant and could simply stop the ciliary beat or retract the velum to sink passively. Fuchs et al. [20] hypothesized that the energetic cost of diving would be offset by fitness gains from raising the probability of hitting discrete patches of oyster reef. Bivalve beds generate more drag and turbulence than surrounding sediments [12], [21], and this roughness-generated turbulence may interact with larval behavior to generate a positive feedback for larval supply to oyster reefs.

We combined empirical models of larval behavior and flow with the “hitting distance” model of McNair and Newbold [14] to estimate the probability that larvae would contact the bottom when passing over patches of different substrate types. This model enabled us to quantify the effects of larval behavior, substrate roughness, current speed, and water depth on larval settlement probabilities and the optimal patch length. The model has applications in oyster restoration and can be used to constrain the size of artificial reef patches over a realistic range of physical habitat characteristics.

## Methods

The “Hitting Distance” model [14] predicts the probability that larvae will contact the bottom within a fixed downstream distance of a starting point at some height above the bed. We used empirical models of larval behavior, currents, and turbulence in the hitting-distance model to characterize settlement of oyster larvae over four different substrates. The empirical models are derived from previously published data [12], [20] and are described briefly below and in Tables S1–S2 and Figures S1–S2.

### Larval Behavior

The larval vertical velocity due to behavior was modeled as an empirical function of dissipation rate based on results of experiments with eyed oyster larvae (*C. virginica*) in grid-stirred turbulence [20]. In that study the larval velocities and water velocities were measured simultaneously using infrared particle-image velocimetry, and behavioral velocities of individual larvae were characterized as a response to the instantaneous dissipation rate 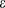. In calm water, larvae swam and rarely sank, but in turbulence they actively dove with greater speed and frequency at higher dissipation rates. Average vertical velocities due to larval behavior ranged from 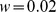 to −1.8 cm s^-1^ over the range 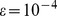 to 10 cm^2^ s^-3^, and the transition from ascending to descending occurred at 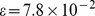 cm^2^ s^-3^. The larval vertical behavioral velocity 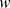 was well described by
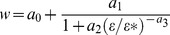
(1)
[20], where 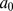 is the maximum ascent velocity, 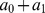 is the maximum descent velocity, 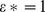 cm ^2^ s^-3^ is a reference value, and the fraction 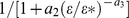 varies from 0 to 1. This model was fitted to the observed behavioral velocities of individual oyster larvae averaged over small bins of dissipation rate to give fitted parameters 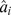 (Table S1).

Active diving is an unusual response to turbulence, and we wanted to quantify how this behavior would affect the probability of larval contact with a reef. To understand the relative effects of active diving on settlement, we used different parameters in Eq. 1 to represent three behaviors: 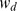 for active diving in strong turbulence, 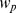 for passive sinking in strong turbulence, and 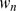 for neutral buoyancy (Table S1, Fig. S1). Parameters for the active diving condition were taken from [20]. Parameters for the passive sinking condition were identical to those for the active sinking condition except that the larval sinking velocity was restricted to the terminal velocity in still water (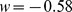 cm s^-1^) [20]. Neutral buoyancy 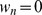 cm s^-1^ is a null model that approximates the behavior of oyster larvae in calm water and represents the expected larval velocities if there were no response to environmental conditions.

### Flow measurements

**Figure 1:**
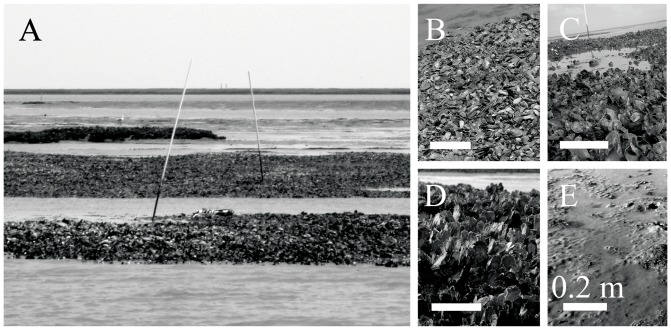
Photos of substrate patches. Sites where flow measurements were made [12], including in order from bottom to top of image (A): oyster shell restoration, whelk shell restoration, mud, and oyster reef. Close-ups are oyster shell (B), whelk shell (C), oyster reef (D), and mud (E). Scale bar on images is 0.2 m.

To model settlement of oyster larvae, we needed realistic profiles of along-stream advection, vertical mixing, and dissipation rate. We used vertical profiles of current speed 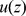, where 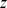 is height above the bed, based on measurements collected at intertidal sites in coastal Virginia [12]. In that study current profiles were measured over four substrate types: natural oyster reef (*C. virginica*), mud, and deposited oyster (*C. virginica*) or whelk (*Busycotypus canaliculatus*) shell (Fig. 1). Substrate patches had surface areas of 270 m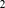 for the natural oyster reef, 1000 m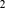 for the mud, 3500 m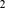 for the oyster shell, and 240 m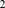 for the whelk shell. All sites were adjacent to one another and located approximately 1 km offshore of Virginia, USA. Flow velocities were measured with a Nortek Aquadopp Profiler in 3 cm increments at heights of 19 to 70 cm above the bottom and averaged over 10-min intervals at different stages of the tide. The shear velocity 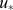 was estimated from each velocity profile by fitting the law of the wall in the log layer.

Flow measurements were collected at discrete depths and at different tidal stages, so we first needed to interpolate, scale, and average the velocity profiles collected over each substrate to create generalized profiles for use in the hitting-distance model. Each measured profile was smoothed using cubic spline interpolation, assuming 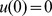 cm s^-1^ at the bottom. To model settlement in deeper water, we extrapolated the smoothed profiles to depths of 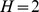 m and 5 m using the log law. Profiles were then normalized by the maximum value 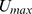, and normalized profiles from each substrate type (12 profiles for natural reefs and 5 profiles for other substrates) were averaged and rescaled by the new maximum value (Fig. S2). For shear velocities we calculated a scale factor 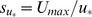 (Table S2). Finally we rescaled the normalized velocity profiles to new maximum velocities of 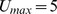 to 100 cm s^-1^ with rescaled shear velocities of 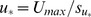. At a given 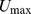 we assumed the flow speed to be constant for the duration of a larval transit over a patch. For each rescaled profile we estimated the vertical eddy diffusivity 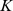 and dissipation rate 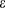 from shear velocity as 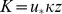 and 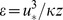, where 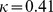 is von Karman's constant.

### Hitting-distance model

The hitting distance model [14] is a differential equation describing the probability 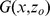 that a particle at initial height 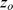 will first hit bottom at a downstream distance 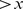, where 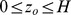 and 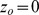 at the bottom. 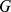 is given by
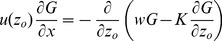
(2)where 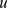 is the along-stream velocity, 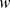 is the larval vertical velocity, and 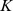 is the vertical eddy diffusivity. The equation is subject to initial conditions at the leading edge of the patch



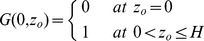
(3)and boundary conditions at the bottom and surface
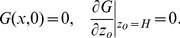
(4)


We incorporated our empirical models in Eqs. 2–4, solved for 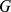 numerically using the Matlab partial differential equation solver, and then differentiated 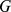 numerically to get 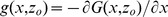, the probability density function for hitting distance. The cumulative distribution of 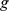 from 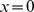 to 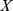 gives 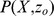, the probability that larvae starting at a height 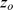 will hit the bottom while passing over a patch of length 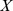. For simplicity we assumed that larvae settle and metamorphose on contact with the bed, so that 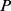 represents a settlement probability. We ran the model using four different substrate types and three different behavior conditions for current speeds of 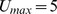 to 100 cm s^-1^, water depths of 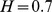, 2.0, and 5.0 m, and patch lengths of up to 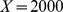 m.

The optimal patch length is defined here as the patch length that jointly maximizes the average settlement probability of individual larvae and the average density of settlers per unit reef length. If the initial vertical distribution of larval concentration is 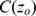, then the depth-averaged probability that larvae will settle within a patch of length 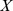 is
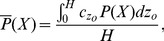
(5)where concentrations are weighted as 
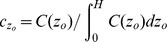
. For this study we assumed that larvae start with an even vertical distribution. Subscripts denote the average settlement probabilities of larvae that dive in turbulence 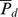, sink passively in turbulence 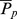, or are neutrally buoyant 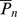, respectively, and these subscripts are used throughout. The average settler density probability per unit reef length is the depth-integrated settlement probability normalized by the patch length,
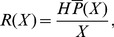
(6)and the average density of recruits per unit reef length is 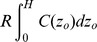. We defined the joint settlement probability 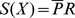 as an indicator of whether individual larvae settle successfully while the reef receives a high density of settlers. The joint settlement probability reached a maximum 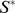 at an optimal patch length 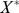 and current speed 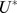.

The maximum joint settlement probability 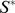 was estimated assuming a constant current speed, but oysters live in tidal systems where the ideal patch characteristics would maximize the average 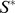 over a complete tidal cycle. The time-averaged 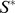 will vary depending on the tidal pattern (e.g., diurnal or semi-diurnal). We estimated optimal patch characteristics for semi-diurnal tides using a simple sinusoidal model of tidal velocity,
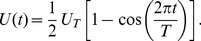
(7)where 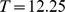 h and the peak tidal velocity 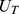 ranged from 1 to 100 cm s^-1^. For each 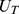 we linearly interpolated the maximum joint settlement probabilities and optimal patch lengths from 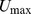 to 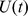 and averaged over the tidal period to get 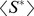 and 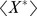, the tidally averaged maximum joint settlement probability and optimal patch length.

## Results

**Figure 2:**
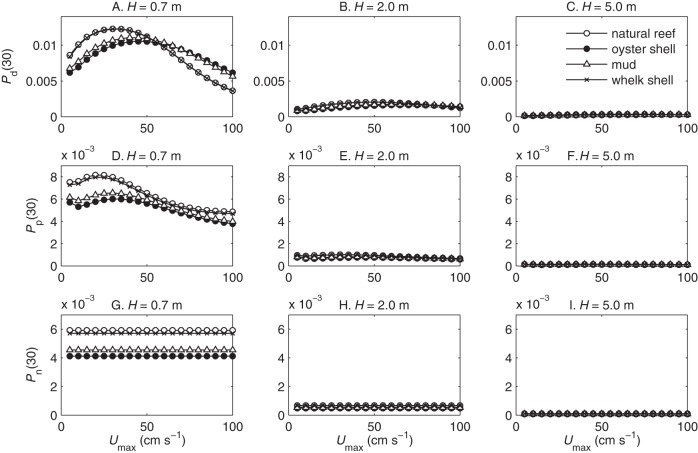
Settlement probability on a 30-m substrate patch. Depth-averaged settlement probabilities vs. current speed 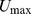 at three water column depths: 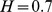 m (A, D, G), 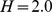 m (B, E, H), and 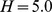 m (C, F, I). Includes settlement probabilities of larvae that dive in turbulence 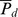 (A–C), larvae that sink passively in turbulence 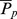 (D–F), and neutrally buoyant larvae 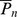 (G–I). Symbols indicate substrate type: open circles, natural reef; closed circles, oyster shell; trianges, mud; 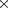, whelk shell.

**Figure 3:**
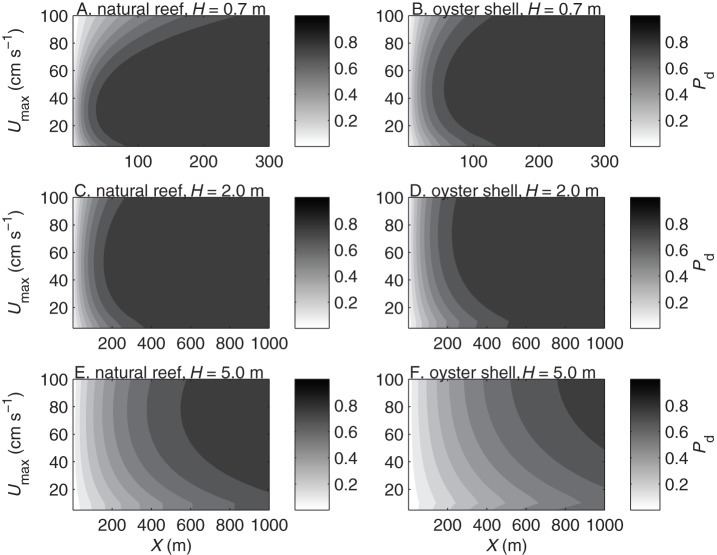
Settlement probability of actively diving larvae. Depth-averaged settlement probability 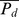 vs. current speed 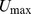 and reef patch length 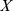 for larvae settling over natural reefs (A, C, E) and deposited oyster shell (B, D, F) in water depths of 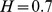 m (A–B), 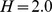 m (C–D), and 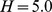 m (E–F).

Depth-averaged larval settlement probabilities 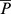 were consistently highest in shallow water, but for diving larvae 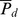 had a dome-shaped relationship with current speed 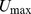 (Fig. 2, 3). The dome shape indicates that the delivery of larvae to the bed is dominated by behavioral vertical advection at low flow speeds and offset by vertical mixing at high flow speeds. An increase in flow speed corresponds both to an increase in dissipation rate, which induces larvae to dive faster, and to an increase in eddy diffusivity, which eventually overwhelms the larval ability to concentrate near the bottom. For passive sinkers, the relationship between settlement probability 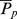 and 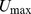 was sometimes bimodal with local maxima both at intermediate current speeds and at the lowest current speeds. Settlement peaked at the lowest current speeds only when turbulence was too weak to induce much larval sinking, so that larvae needed a long travel time over the patch to contact the bed. Neutrally buoyant larvae always had an even vertical distribution and settlement probabilities 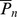 that were independent of flow speed.

Settlement probabilities were highest over rougher substrates, which produced more turbulence that contributed both to dissipation-induced larval descents and to vertical mixing. Diving larvae had higher peak settlement probabilities at lower optimal flow speeds over hydraulically rougher substrates (natural reef and whelk shell) than over smoother substrates (mud and oyster shell). One counter-intuitive result was that settlement probabilities over mud were slightly higher than those over deposited oyster shell (Fig. 2). Currents over the mud patch were measured near (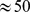 m distant) the natural oyster reef and restoration sites and probably retained some influence of the nearby reef. The natural reef and deposited whelk shell also had similar results due to their similar ratio of current speed to shear velocity 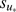. Hereafter we report results only for natural oyster reefs and deposited oyster shell.

**Figure 4:**
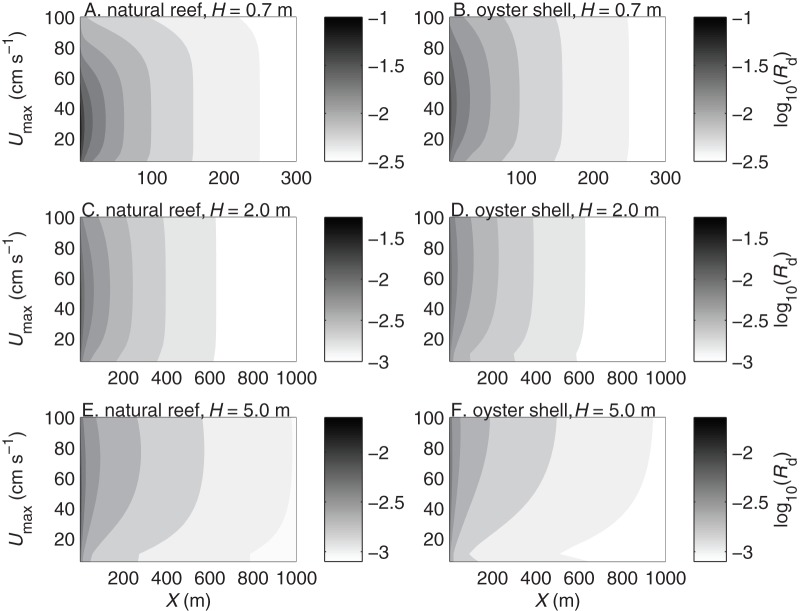
Settler density probability of actively diving larvae. Settler density probability per meter of reef length 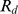 vs. current speed 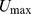 and reef patch length 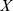 for larvae settling over natural reefs (A, C, E) and deposited oyster shell (B, D, F) in water depths of 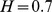 m (A–B), 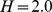 m (C–D), and 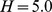 m (E–F). Settler density probability is shown on a 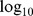 scale.

The settlement probability 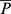 generally increased with patch length (Fig. 3), whereas the settler density probability 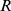 decreased with patch length (Fig. 4). The patch length required for most larvae (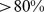) to settle was shorter in shallower water than in deeper water and shorter over rougher substrates than over smoother substrates (Fig. 3). Although individual larvae are more likely to contact a large patch than a small one, the settlement probability increases with patch length most rapidly at small 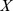. Thus most larvae would settle near the leading edge of a patch, and increasing the patch length would reduce the average density of settlers per unit patch length. The settler density probability 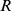 was higher in shallower water than in deeper water and slightly higher over rougher substrates than over smoother substrates (Fig. 4). Substrate type had little effect on settler density except on the smallest patches in weak currents, where 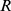 was up to 1.8 times higher on the natural oyster reef than on deposited oyster shell. The settler density probability, like the settlement probability, had a dome-shaped relationship with 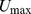 and was highest at intermediate flow speeds.

**Figure 5:**
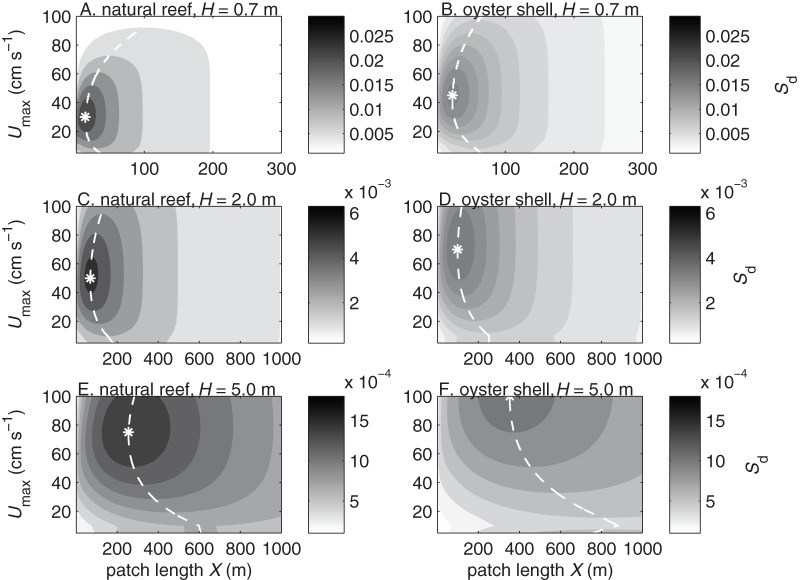
Joint settlement probability of actively diving larvae. Joint settlement probability 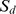 vs. current speed 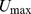 and reef patch length 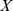 for larvae settling over natural reefs (A, C, E) and deposited oyster shell (B, D, F) in water depths of 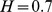 m (A–B), 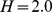 m (C–D), and 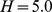 m (E–F). White dashed lines indicate optimal patch lengths 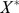 at each current speed, and 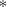 indicates the overall optimal patch length 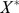 and current speed 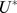 where joint settlement probability reaches a maximum 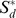.

**Figure 6:**
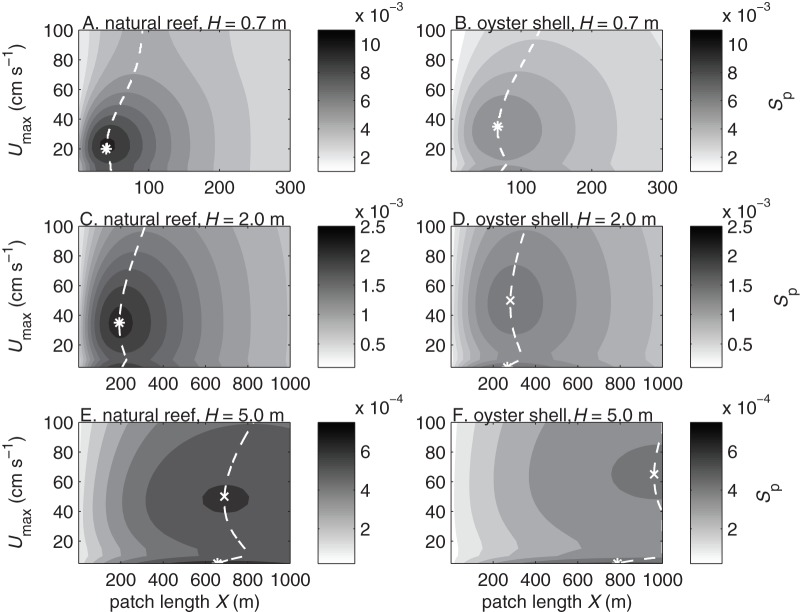
Joint settlement probability of passively sinking larvae. Joint settlement probability 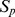 vs. current speed 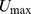 and reef patch length 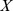 for larvae settling over natural reefs (A, C, E) and deposited oyster shell (B, D, F) in water depths of 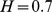 m (A–B), 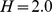 m (C–D), and 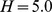 m (E–F). White dashed lines indicate optimal patch lengths 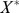 at each current speed, 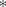 indicates the overall optimal patch length 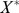 and current speed 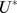 where joint settlement probability reaches a maximum 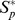, and 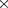 indicates second local maximum 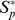.

**Figure 7:**
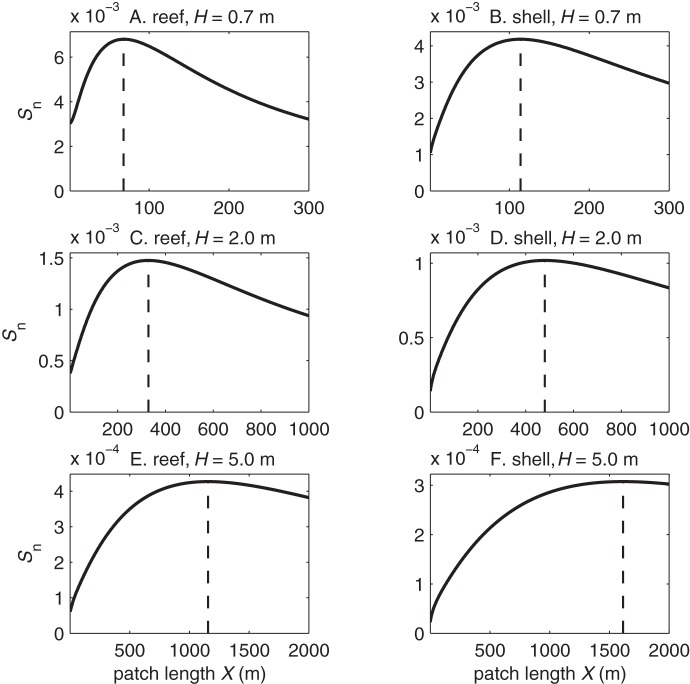
Joint settlement probability of neutrally buoyant larvae. Joint settlement probability 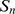 vs. reef patch length 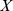 for larvae settling over natural reefs (A, C, E) and deposited oyster shell (B, D, F) in water depths of 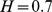 m (A–B), 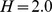 m (C–D), and 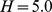 m (E–F). Dashed lines indicate optimal patch lengths 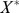 where joint settlement probability reaches a maximum 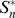.

The joint settlement probability 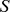 had a maximum value 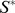 at an optimal current speed 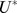 and patch length 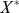 (Fig. 5, 6, 7). For diving larvae, the maximum 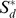 occurred at a higher 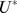 and a longer 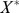 in deeper water than in shallower water and over smoother substrates than over rougher substrates. Passively sinking larvae had similar results except that like 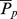 the joint settlement probability sometimes had two local maxima, where the largest 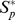 occurred at the lowest current speed (Fig. 6D–F). The peak at the lowest current speed indicates that in nearly stagnant currents the dissipation rates were high enough near the bed to induce some sinking, while the weak vertical mixing and slow currents gave larvae a long travel time over the reef bed and enabled high settlement rates. For neutrally buoyant larvae 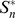 was independent of current speed but varied with patch length, and 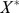 was longer in deeper water than in shallower water (Fig. 7).

**Figure 8:**
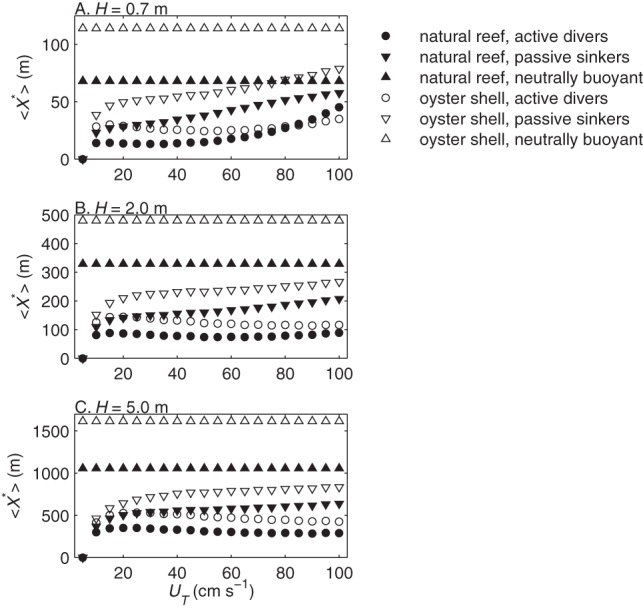
Tidally averaged optimal patch lengths. Optimal patch length averaged over a tidal cycle 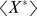 vs. peak tidal velocity 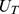 at water depths of 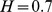 m (A), 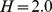 m (B), and 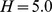 m (C). Symbols indicate behavior and substrate type: circle, diving larvae; down-triangle, passively sinking larvae; and up-triangle, neutrally buoyant larvae on natural oyster reef (closed symbols) and oyster shell (open symbols).

**Figure 9:**
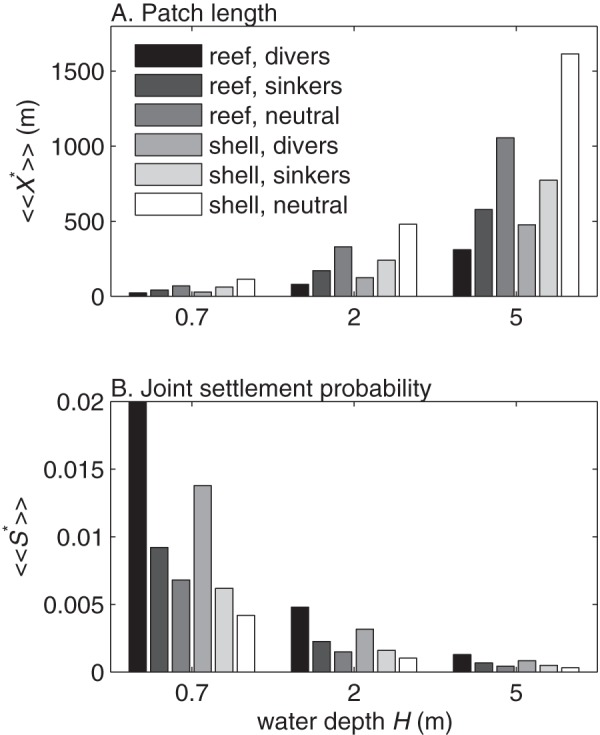
Mean optimal patch lengths. Mean of tidally averaged A) optimal patch length 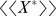 and B) maximum joint settlement probability 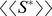 vs. water depth 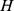. Means are computed from tidally averaged values with peak tidal velocities 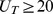 cm s^-1^. Results are shown for diving larvae, passively sinking larvae, and neutrally buoyant larvae on natural oyster reef and deposited oyster shell.

The tidally averaged optimal patch lengths 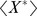 varied little with peak tidal velocities except in relatively slow currents (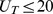) but varied considerably with water depth, larval behavior, and substrate type (Fig. 8). To quantify this variation we averaged the optimal patch lengths and joint settlement probabilities for all 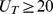 cm s^-1^ to get mean tidally averaged values 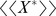 and 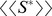 (Fig. 9). Mean optimal patch lengths ranged from 22 m for diving larvae in 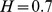 m depth to 1615 m for neutrally buoyant larvae in 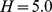 m depth. For a given combination of substrate and behavior, varying the water depth from 0.7 to 5 m accounted for up to a factor of 17 variation in 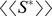 and 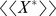. For all substrate types or water depths, the joint settlement probability 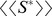 of diving larvae were about double those of passive sinkers and about three times those of neutrally buoyant larvae. Neutrally buoyant larvae had optimal patch lengths 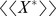 1.5 to 2 times greater than those of passive sinkers and 3 to 4 times greater than those of diving larvae for all substrate types and water depths. Joint settlement probabilities 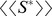 were 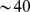 to 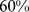 higher on natural oyster reef than on deposited shell, whereas optimal patch lengths 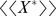 were 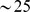 to 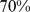 larger for deposited oyster shell than for natural reefs. Overall, substrate roughness had a relatively small effect on settlement probabilities, settler densities, and optimal patch length when compared to water depth or larval behavior.

## Discussion

Larval settlement is critical for the formation and maintenance of oyster reefs, and this study quantifies settlement variation arising from larval behavior and physical habitat characteristics. Although behavior is species-specific, the habitat characteristics we addressed are universal, and our approach and results are generally applicable for other reef-building species. In particular the reef length can be optimized by considering the tradeoff between higher settlement probabilities on large patches and higher recruitment densities on small patches. A patch shorter than the tidally averaged optimal 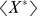 will achieve a high settler density at the cost of a low larval settlement probability, wasting more larvae. A patch longer than 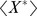 will achieve a high larval settlement probability at the cost of a low settler density, limiting upward reef growth. The optimal 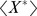 is a target size for patch restoration. The value of 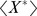 depends on larval behavior and physical habitat characteristics about which some uncertainty is inevitable. Since the joint settlement probability 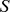 is most variable at small 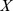 (e.g., Fig. 7), it would be better to err on the side of creating larger patches. Predictions from this model are testable and suggest new guidelines for improving the design of restored oyster reefs.

### Importance of habitat characteristics

The prediction that optimal patch size increases with water depth is consistent with natural variation in the size of oyster reefs. For example, in the relatively shallow Duplin River (Georgia) there are hundreds of small oyster beds on the edges of the channel (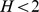 m) with patch lengths of 10 s to 100 s of m [22], [23]. The James Estuary in southern Chesapeake Bay (Virginia) historically had oyster reefs in intermediate depths (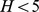 m) with patch lengths of a few 100 s of m to 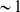 km [24], [25]. In the deeper Delaware Bay (New Jersey), oyster beds in the shallower waters (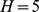 to 10 m) of the upper bay and near shore are smaller (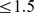 km) whereas those in deeper water (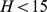 m) toward the main channel and west end of the bay are larger (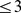 km) [5]. Data on patch lengths are sparse and imprecise but suggest that natural reefs are generally within the range of optimal sizes predicted by the hitting-distance model.

Although patch size and water depth are relatively easy to observe, recruitment rates are measured in diverse ways that make comparisons to model results difficult. In particular the model predicts only settlement and settler-density probabilities, whereas observations quantify total recruitment that reflects additional variation in larval concentrations and post-settlement mortality. It is also difficult to compare time-integrated recruitment on subtidal reefs and intertidal reefs that are submerged for only a fraction of the tidal cycle. Observations provide conflicting evidence of whether recruitment rates are higher on subtidal or intertidal reefs [7], [26], [27], and these inconsistencies may arise from differences in larval supply, submergence time, or reef size itself.

One unexpected result of this study was that when compared to water depth or larval behavior, substrate roughness accounted for the least variability in settlement or optimal patch lengths. Many comparative or manipulative studies have focused on how substrate composition or shape affect recruitment rates on restored reefs. Although substrate types affect post-settlement survival [12] and should still be chosen with care, our results suggest that greater effort should be devoted to understanding other sources of recruitment variability. In fact the simplest and most effective way to optimize the design of restored reefs may be to ensure that deposited substrate patches are of an appropriate size for the average water depth.

These results point to an underappreciated potential mechanism for the global collapse of historical oyster reefs [28], [29]. When reef substrate is removed by destructive harvesting [6], [30], the loss of broodstock oysters reduces the potential total reproductive output while shrinking the reef footprint. If a reef patch was near the optimal size before harvesting, then any reduction in patch size would also reduce the settlement probability of individual larvae (Fig. 3) and the maximum attainable joint settlement probability (Figs. 5, 6, 7). Through its effects on settlement, a loss of reef habitat may ultimately increase larval wastage and reduce the effective fecundity of remaining adults.

### Importance of larval behavior

Model results imply that larval diving in turbulence confers advantages besides higher settlement rates. Sinking by late-stage larvae generally enhances larval retention near estuarine habitats [31], [32] and near-bed concentrations in tidal currents [17]. Oyster larvae are negatively buoyant and could achieve those benefits by sinking passively. Compared to passive sinkers in this model, however, diving larvae had about twice the joint settlement probability and about half the optimal patch length. Smaller reef patches are beneficial for filter feeders because suspended particles become depleted over the length of a patch [33], and adult oysters on small reefs will receive more food on average than those on large reefs. Thus turbulence-induced diving not only enhances the probability of larvae hitting a reef, it also provides a potential mechanism to shorten reef lengths and enhance the average food supply for adults. These combined benefits could offset the energetic costs of diving in turbulence.

The modeled effects of larval behavior on settlement were non-trivial, suggesting that for species whose economic value relies on habitat restoration, greater attention should be paid to characterizing larval behaviors. Here we ignored other behavior cues and focused on how settlement is affected by responses to turbulence as observed in the laboratory [20]. Behavior is also modified by salinity and temperature, although the absolute larval swimming speed changes by only a few percent per unit salinity or degree C [34], [35]. Responses to salinity and temperature would have little effect on net motion, because swimming larvae alternate between ascending and descending and have average vertical swimming speeds near zero [20], [34]. In contrast, turbulence induces a change in behavior mode that can alter larvae vertical velocities by centimeters per second over a realistic range of turbulence conditions, and for some species the overall behavior in situ appears to be dominated by responses to turbulence [36]. It remains to be tested whether laboratory observations on oyster larvae resemble behavior in estuaries where turbulence is anisotropic, topographically influenced, and potentially conflated with waves.

Perhaps the largest uncertainty for oyster larval behavior is how vertical velocities and attachment probabilities are influenced by chemical cues. Settlement is enhanced by chemicals from adult oysters [13], [37], but the interactions between chemical cues and flow are less well understood for oysters than for other species. Nudibranch larvae *Phestilla sibogae* sink and attach to substrates when they encounter a chemical cue from their obligate coral prey species [38], [39], and this behavior can enhance transport to and settlement success on coral reefs in wavy, turbulent flow [40], [41]. Chemical cues diffuse rapidly and may be detectable only within a few cm of the substrate [40], [42], so cue effectiveness depends on turbulence intensity. For oyster larvae the combined responses to chemical cues and turbulence on oyster reefs may produce complex settlement dynamics.

Until behavioral uncertainties are resolved, our model results can be viewed as a range of potential outcomes for oyster settlement. The true joint settlement probability and optimal patch size should lie somewhere between those estimated here for diving larvae and for neutrally buoyant larvae. Between these two extremes of behavior, the mean 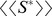 and 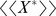 varied by a factor of three to four, indicating that settlement dynamics are sensitive to behavioral effects on larval delivery to the bed. This sensitivity to larval behavior is pervasive in larval transport models, and our results underscore the importance of using appropriate empirical models of how larvae respond to the environment.

### Model limitations

The hitting-distance model has only one horizontal spatial dimension and a homogeneous bottom, limiting our ability to assess the importance of patch shape or elevation. Oyster beds have various 2-dimensional shapes ranging from relatively round reefs to more string-shaped reefs aligned either with or across the main current [3]. Settlement should be highest near the leading edge, but the leading edge alternates with the tidal stage so that settlement is distributed over both ends of the patch. Symmetric and asymmetric tides may produce different optimal shapes, and round reefs may form when the magnitude of cross-current flow is high relative to along-current flow. The omission of bottom topography obscures other physical effects on settlement from reef elevation or availability of interstitial spaces [4], [12], [41] that require further investigation. This model suggests guidelines for the maximum dimension of a restored reef in the along-current direction, whereas the patch shape should be modified according to local cross-current flow conditions.

The model's lack of time dependence makes it difficult to account for tidal variation in current velocities and water depths. Instead we assumed that current speeds are constant for the duration of larval travel over the patch and that an average water depth is representative of the variation in tidal height. The current speed assumption is reasonable under most conditions but becomes less tenable over large reefs. We expect that this assumption contributes only small errors, because the peak tidal velocity had little influence on tidally averaged results (Fig. 8). Larger errors may arise from the use of a constant water depth, given that settlement varied greatly with depth. Future studies could account for tidal variation in water depth the same way we accounted for tidal variation in velocity, by generating model results for a more highly resolved range of depths and velocities and interpolating results over a tidal cycle with changing tidal heights. That approach would be computationally expensive and is better suited for predicting optimal patch lengths in a specific system, particularly where reefs are submerged for only a fraction of the tidal cycle.

Optimal patch sizes may also be affected by other processes that contribute to oyster fitness. Food particles are delivered to adult oysters by the same physical processes that supply larvae to a reef, so food supply is proportional to 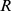 for arriving food particles. Assuming that food particles are neutrally buoyant, we could account for food supply by maximizing 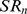, which would reduce the estimated optimal patch size. Although a smaller reef may receive more food per unit length, it would also be more susceptible to hazards at the reef edge such as predation and sedimentation [2]. These tradeoffs are better understood for mussel beds, which experience both greater food ability and higher dislodgment forces at the patch edge [33], [43], [44]. Like mussel beds, oyster reefs are further constrained by topography and other landscape characteristics [45], and these processes should be addressed in system-specific studies.

### Scaling up from patches to metapopulations

Larvae can settle successfully only if they reach competency and encounter a patch while dispersing, and it is unclear how patches should be arranged in an estuary or region to globally optimize the success of both dispersing larvae and individual reef patches. For a single patch the optimal habitat configuration is independent of larval concentration, but in a metapopulation the relative success of individual patches will depend on larval supply and the distribution of source and sink populations [2], [46]. The optimal distribution of patches may also depend on the dispersal distance during the larval development time or the degree of isolation needed to minimize the spread of disease or predators among patches [2]. To better meet management goals, this and other optimization approaches should be combined in a spatially explicit metapopulation framework [47]–[49].

## Supporting Information

Figure S1Larval behavior functions. Larval behavioral vertical velocity vs. dissipation rate 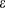 for three behaviors used: active diving in turbulence 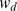 (solid line) [20], passive sinking in turbulence 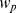 (dashed line), and neutral buoyancy 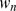 (dotted line).(EPS)

Figure S2Current velocity profiles. Height 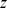 vs. normalized mean current velocities 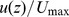 over four substrates: healthy oyster reef, deposited oyster shell, deposited whelk shell, and mud. A–D) Velocity profiles interpolated from observations in 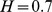 m depth [12] and normalized by the maximum velocity, including individual profiles (red lines) and their mean (black lines). Remaining panels as in A–D except interpolated profiles were extrapolated to a height of 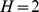 m (E–H) or 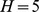 m (I–L) using the log law before being normalized by maximum velocity.(EPS)

Table S1 (PDF)

Table S2 (PDF)
