# Correction: Biophysical Constraints on Optimal Patch Lengths for Settlement of a Reef-Building Bivalve

**DOI:** 10.1371/annotation/30af0dbd-0e47-4ea6-a2b8-4255ff782d1f

**Published:** 2013-11-07

**Authors:** Heidi L. Fuchs, Matthew A. Reidenbach

Typographical errors occurred in References 4, 11, 12, 16, 25, 27, 31, 33, and 49. Please see the correct References section here: http://www.plosone.org/corrections/pone.0071506.001.cn.pdf

