# Supplementary material for: Biophysical Constraints on Optimal Patch Lengths for Settlement of a Reef-Building Bivalve
Source: PLoS One. 2013 Aug 19;8(8):e71506. doi: 10.1371/journal.pone.0071506 (PMC3747277; doi:10.1371/journal.pone.0071506)
Supplement: Table S1 — (PDF) [file pone.0071506.s003.pdf]

Table S1: Parameters used in behavior model.

Behavior	$\hat{a}_0$ (cm s^{-1})	$\hat{a}_1$ (cm s^{-1})	$\hat{a}_2$	$\hat{a}_3$
w_d	0.023	-3.66	10.8	1.05
w_p	0.023	-0.61	10.8	1.05
w_n	0	0	0	0

Parameter sets $\hat{a}_i$ were used in Eq. 1 to model settlement of larvae with three different behaviors: w_d active diving in turbulence [20], w_p passive sinking in turbulence, and w_n neutral buoyancy.
