# Supplementary material for: Biophysical Constraints on Optimal Patch Lengths for Settlement of a Reef-Building Bivalve
Source: PLoS One. 2013 Aug 19;8(8):e71506. doi: 10.1371/journal.pone.0071506 (PMC3747277; doi:10.1371/journal.pone.0071506)
Supplement: Table S2 — (PDF) [file pone.0071506.s004.pdf]

Table S2: Characteristics of four substrate types.

Substrate	$\bar{z}_{\mathrm{o}}$ (cm)	C_{D}	s_{u_*}
Natural reef	1.5	0.019	0.114
Oyster shell	0.3	0.008	0.075
Whelk shell	1.7	0.011	0.119
Mud	0.4	0.009	0.082

Average roughness height $\bar{z}_{\mathrm{o}}$, drag coefficient C_{D}, and shear velocity scale factor s_{u_*} for velocity profiles used in the hitting-distance model. Measurements described in [12].
